# Operating-room temperature and warming protocols as predictors of postoperative hypothermia in ovarian cancer surgery: A retrospective cohort study

**DOI:** 10.1097/MD.0000000000047452

**Published:** 2026-01-30

**Authors:** Lu Mo, Jing Fu, Wei Zhang, Jingyue Kuang, Tao Jiang

**Affiliations:** a Department of Operating Room, The Affiliated Hospital, Southwest Medical University, Luzhou, Sichuan, China; b Outpatient Department, The Affiliated Hospital, Southwest Medical University, Luzhou, Sichuan, China.

**Keywords:** cytoreductive surgery, hypothermia, ovarian cancer, perioperative nursing, warming protocols

## Abstract

Perioperative hypothermia is a common but preventable complication associated with increased morbidity and delayed recovery in surgical oncology patients. However, limited evidence exists regarding its prevalence, predictors, and consequences in women undergoing cytoreductive surgery for ovarian cancer. This retrospective cohort study included 245 patients who underwent primary or interval cytoreductive surgery for epithelial ovarian cancer at a tertiary care center between 2014 and 2022. Patients were stratified into normothermia (≥36.0°C) and hypothermia (<36.0°C) groups based on core temperature at surgical closure. Intraoperative warming strategies and operating-room temperature were recorded. Multivariate logistic regression was used to identify independent predictors of postoperative hypothermia. Postoperative outcomes were compared between groups. Postoperative hypothermia occurred in 39.6% of patients. Independent risk factors included age ≥ 60 years (odds ratio [OR] 1.89, *P* = .025), body mass index < 22 kg/m² (OR 1.96, *P* = .022), American Society of Anesthesiologists class III–IV (OR 2.05, *P* = .020), ascites volume > 500 mL (OR 1.97, *P* = .031), operative time ≥ 240 minutes (OR 2.92, *P* = .001), blood loss ≥ 400 mL (OR 2.23, *P* = .011), absence of active warming (OR 4.12, *P* < .001), and OR temperature < 22°C (OR 2.47, *P* = .005). Hypothermia was associated with higher rates of shivering (40.2% vs 12.2%, *P* < .001), surgical site infection (16.5% vs 6.8%, *P* = .019), longer time to ambulation and gastrointestinal recovery, prolonged hospital stay, and increased 30-day readmission. Postoperative hypothermia is highly prevalent and clinically significant among ovarian cancer patients undergoing cytoreductive surgery. Intraoperative warming strategies and maintaining adequate OR temperatures play critical roles in prevention. These findings highlight the importance of standardized thermal care protocols led by perioperative nursing teams to improve surgical outcomes. These conclusions apply to female patients only, as the cohort exclusively comprised women undergoing ovarian cancer cytoreductive surgery.

## 1. Introduction

Inadvertent perioperative hypothermia – defined as a core body temperature below 36.0°C – is a frequent complication during major surgical procedures, affecting up to 80% of cases despite preventive efforts.^[1,2]^ Hypothermia increases the risk of surgical site infection (SSI), impairs coagulation, prolongs recovery, and increases healthcare costs.^[3-6]^ International guidelines, including those from AORN and ASPAN, recommend active warming strategies and maintaining odds ratio (OR) temperatures ≥ 21°C to mitigate these risks.^[7-9]^

While extensive data support these interventions in general surgical populations, their application in gynecologic oncology – particularly ovarian cancer cytoreductive surgery – remains understudied. Such procedures often involve prolonged exposure, high fluid volumes, and extensive dissection, all contributing to thermoregulatory instability.^[10,11]^ Prior retrospective cohorts reported intraoperative hypothermia rates between 45% and 72% in ovarian cancer surgery.^[12,13]^ However, these studies did not comprehensively assess nurse-led intraoperative warming protocols or ambient OR conditions.

Moreover, current evidence largely originates from non-oncology settings or is limited by weak methodology. For example, although nurse-led warming initiatives have demonstrated efficacy in larger perioperative populations,^[14,15]^ these interventions seldom appear in gynecologic cancer protocols. Nursing-led studies in other contexts have reinforced the value of structured warming practices but often lack integration with OR environmental controls.^[16,17]^

To address these gaps, we performed a retrospective study involving 245 patients undergoing cytoreductive surgery for ovarian cancer at a tertiary academic center (2014–2022). This research investigates the incidence of postoperative hypothermia, explores predictors such as intraoperative warming protocols (nurse-initiated prewarming and forced-air warming) and OR ambient temperature, and evaluates associations with recovery outcomes, including shivering, SSI, gastrointestinal and mobilization metrics, length of stay, and readmission rates. We hypothesized that standardized nurse-led thermal protocols and maintaining OR temperatures ≥ 22°C would significantly reduce hypothermia rates and enhance postoperative recovery.

Distinctively, this study brings a perioperative nursing perspective by examining nurse-initiated warming practices, OR team collaboration, and integration with postanesthesia care, thereby aligning with current efforts to implement evidence-based, nurse-driven thermal management in complex oncologic surgery. Our findings aim to inform the development of strategic, standardized thermal nursing protocols in gynecologic oncology operating rooms, ultimately improving patient outcomes and safety.

Ovarian cytoreductive surgery involves extensive peritoneal exposure, multi-quadrant debulking, diaphragmatic stripping, and frequent evacuation of large-volume ascites – procedures that substantially increase conductive and evaporative heat loss.^[18]^ Recent multicenter gynecologic studies have reported intraoperative hypothermia incidences of approximately 45% across gynecologic surgeries,^[19]^ with even higher risks documented among patients undergoing ovarian cancer cytoreduction because of prolonged operative times and extensive visceral exposure.^[20]^ In comparison, large noncardiac abdominal surgery cohorts generally report postoperative hypothermia incidences of around 30% despite similar procedure durations and blood loss.^[21]^ Collectively, these findings indicate that ovarian cancer cytoreduction represents a particularly high-risk surgical setting for perioperative hypothermia, underscoring the importance of evaluating modifiable thermal management measures such as active warming and operating-room temperature control.

## 2. Materials and methods

### 2.1. Study design and reporting standards

This was a retrospective cohort study conducted at the Department of Gynecologic Oncology, The Affiliated Hospital, Southwest Medical University (Luzhou, China), a tertiary care center with a stable surgical and perioperative nursing team during the study period. The study period spanned from January 2014 to December 2022. We deliberately restricted inclusion to epithelial ovarian cancer cytoreductive surgeries to minimize procedural heterogeneity and to study a population with a priori high hypothermia risk due to extensive peritoneal exposure and fluid shifts. This single tertiary center performs approximately 25 to 30 cytoreductive surgeries for epithelial ovarian cancer annually, reflecting a stable case volume across the study period. The 9-year inclusion window therefore represents consecutive accrual rather than selective sampling. We intentionally did not include other gynecologic or general abdominal procedures performed at the same institution because their surgical scope, exposure extent, and anesthesia–nursing thermal workflows differ substantially. Pooling these heterogeneous surgeries would have increased sample size but introduced confounding, thereby compromising internal validity for identifying modifiable thermal factors such as active warming and operating-room temperature. Consequently, the cohort comprises female patients only; generalizability is discussed below.

### 2.2. Surgical setting and environmental control

All procedures were conducted within the same institution in a set of dedicated gynecologic oncology operating rooms located in a single surgical suite. These rooms are served by a centralized HVAC system with harmonized thermostat calibration and scheduled maintenance according to institutional policy.

### 2.3. Patient selection and grouping

Patients were eligible if they: had histologically confirmed epithelial ovarian cancer; underwent primary or interval cytoreductive surgery; were aged ≥ 18 years; and had complete perioperative records, including intraoperative temperature monitoring and postoperative recovery data. Exclusion criteria included: synchronous malignancies; incomplete surgical or anesthesia records; preoperative hypothermia or febrile conditions; and conversion to non-radical or palliative procedures intraoperatively.

Patients were classified into 2 groups based on their core body temperature at the end of surgery: normothermia (≥36.0°C) and hypothermia (<36.0°C), in accordance with the definitions recommended by the American Society of PeriAnesthesia Nurses (ASPAN) and supported by prior literature. Core temperature was continuously monitored using calibrated esophageal temperature probes and documented every 15 minutes. Only the final recorded temperature at surgical closure was used for classification to ensure consistency.

### 2.4. Data collection and variable definitions

Demographic data (age, body mass index [BMI]), clinical characteristics (American Society of Anesthesiologists [ASA] physical status, International Federation of Gynecology and Obstetrics stage), preoperative laboratory results (hemoglobin, albumin), and surgical data (operative time, blood loss, ascites volume, and completeness of cytoreduction) were extracted from the hospital’s electronic medical record system by 2 trained abstractors. Discrepancies were resolved by a third investigator.

Intraoperative warming strategies were categorized as: forced-air warming only; prewarming combined with forced-air warming; and no active warming. The presence of any warming device was recorded in the anesthesia nursing log. Operating room ambient temperature was recorded hourly via the central temperature control system and confirmed by circulating nurses. Surgeries were performed in different gynecologic oncology operating rooms within the same suite; for each case, the room identifier and hourly ambient temperature were documented. For analysis, the actual recorded ambient temperature was used as the exposure variable rather than room identity, given the standardized HVAC controls across rooms. For analytical purposes, OR temperature was categorized as <21°C, 21.0 to 22.9°C, or ≥23°C, consistent with institutional infection control and safety protocols.

### 2.5. Anesthesia management variables

Anesthetic data were abstracted from the anesthesia record. Anesthesia maintenance mode was categorized as volatile-based general anesthesia (inhalational agent with opioid and neuromuscular blockade) versus total intravenous anesthesia (TIVA; propofol/remifentanil-based), and the use of neuraxial adjuncts (epidural or spinal/epidural catheter for analgesia; yes/no) was recorded. Intraoperative vasoactive/vasoconstrictor use (phenylephrine and/or ephedrine administered for anesthesia-related hypotension) was captured as a binary variable (any use vs no use) due to inconsistent dose/timing documentation across years. These anesthesia-related variables were included as candidate covariates in univariable screening and were eligible for multivariable selection when *P* < .10 and/or clinically justified.

### 2.6. Intraoperative vasoactive medications

From the anesthesia record we abstracted the use of vasoconstrictor/vasopressor agents (phenylephrine and/or ephedrine) administered to treat anesthesia-related hypotension. Because cumulative dose and precise timing were inconsistently documented across years, vasoactive exposure was coded as a binary variable (any use vs no use) for analysis.

Postoperative outcomes included: shivering in the postanesthesia care unit (PACU), as documented by PACU nurses; SSIs, defined using Centers for Disease Control and Prevention (CDC) criteria, including evidence of purulent drainage, erythema with incision opening, or physician diagnosis; time to first flatus and ambulation (in hours post-surgery); length of postoperative hospital stay (in days); and 30-day unplanned readmissions, verified through discharge and follow-up records.

### 2.7. Statistical analysis

All analyses were performed using SPSS version 26.0 (IBM Corp., Armonk). Normality of continuous data was assessed with the Shapiro–Wilk test. Data are presented as mean ± standard deviation or median and interquartile range (IQR), as appropriate. Group comparisons were made using independent *t*-tests or Mann–Whitney *U* tests for continuous variables and Chi-square or Fisher’s exact tests for categorical variables.

Univariate analyses were first conducted to identify potential risk factors associated with postoperative hypothermia. Variables with *P* < .10 and those deemed clinically relevant were entered into a multivariate logistic regression model using backward stepwise selection. Anesthesia maintenance mode (volatile-based vs TIVA), neuraxial adjuncts (yes/no), and vasoactive use (any vs none) were included among candidate predictors in the univariable screening set and considered for multivariable entry based on the same criteria. Adjusted ORs with 95% confidence intervals (CIs) were reported. Model calibration was assessed using the Hosmer–Lemeshow goodness-of-fit test (*P* > .05 indicating good fit), and multicollinearity was checked using the variance inflation factor, with variance inflation factor > 10 considered indicative of significant collinearity. Laboratory variables (hemoglobin, albumin) had low missingness (<5%) and were handled using complete-case analysis. Candidate covariates with *P* < .10 in univariable testing and/or strong clinical relevance were entered into the multivariable model with backward selection while maintaining model parsimony and events-per-variable thresholds. Vasoactive use (phenylephrine/ephedrine; any vs none) was included among candidate covariates in univariable screening and entered into the multivariable selection set when *P* < .10 and/or clinically justified. Hemoglobin and albumin were explored in univariable analyses but were not retained in the final model after multivariable screening (see Table S1, Supplemental Digital Content, https://links.lww.com/MD/R269).

As this was a retrospective, population-based cohort study, no a priori sample size calculation was performed. However, the final sample size (n = 245) met the recommended event-per-variable threshold for regression analysis. To minimize information bias, all data were double-entered and validated, and variable thresholds (e.g., BMI < 22 kg/m², operative time ≥ 240 min) were defined a priori based on clinical relevance and cohort distribution.

## 3. Results

### 3.1. Baseline characteristics of the study population

A total of 245 ovarian cancer patients who underwent cytoreductive surgery between 2014 and 2022 were included in the study. Among them, 97 (39.6%) developed postoperative hypothermia, while 148 (60.4%) maintained normothermia. As shown in Table 1, patients in the hypothermia group were significantly older (57.6 ± 9.9 vs 54.0 ± 9.5 years, *P* = .008) and had lower BMI (21.8 ± 3.7 vs 23.1 ± 3.1 kg/m², *P* = .003). Higher ASA classification (III–IV) was more prevalent in the hypothermic group (*P* = .038), as was advanced International Federation of Gynecology and Obstetrics stage III–IV (*P* = .009). Surgically, the hypothermia group had significantly longer operative durations (239.1 ± 55.4 vs 204.5 ± 48.3 minutes, *P* < .001), greater intraoperative blood loss (400 [280–600] vs 250 [180–400] mL, *P* < .001), and more frequent ascites volumes > 500 mL (47.4% vs 25.0%, *P* = .001). Additionally, the normothermia group had a significantly higher rate of complete macroscopic cytoreduction (R0) compared to the hypothermia group (72.3% vs 56.7%, *P* = .015). Preoperative laboratory parameters also differed significantly between groups: patients who developed hypothermia had lower hemoglobin (115.2 ± 13.5 vs 118.6 ± 12.7 g/L; *P* = .045) and albumin levels (37.6 ± 4.2 vs 39.1 ± 3.9 g/L; *P* = .013) than those who maintained normothermia (Table 1). Regarding anesthetic management, the distribution of anesthesia maintenance mode (volatile-based general anesthesia vs TIVA) and the use of neuraxial adjuncts was comparable between the hypothermia and normothermia groups (both *P* > .20; see Table S1, Supplemental Digital Content, https://links.lww.com/MD/R269), indicating no crude group imbalance in these parameters. Notably, intraoperative warming methods and operating-room (OR) temperature were significantly associated with thermal outcomes: patients in the normothermia group were more likely to have received active warming and were exposed to higher OR temperatures (*P* < .001 for both).

**Table 1:** Baseline characteristics of ovarian cancer patients undergoing cytoreductive surgery at The Affiliated Hospital of Southwest Medical University (2014–2022).

Variable	Total (n = 245)	Normothermia (n = 148)	Hypothermia (n = 97)	*P*-value
Patient characteristics				
Age, yr (mean ± SD)	55.3 ± 9.8	54.0 ± 9.5	57.6 ± 9.9	.008**
BMI, kg/m² (mean ± SD)	22.6 ± 3.4	23.1 ± 3.1	21.8 ± 3.7	.003**
ASA classification, n (%)				.038*
I–II	176 (71.8%)	114 (77.0%)	62 (63.9%)	
III–IV	69 (28.2%)	34 (23.0%)	35 (36.1%)	
FIGO stage III–IV, n (%)	184 (75.1%)	102 (68.9%)	82 (84.5%)	.009**
Preoperative hemoglobin, g/L (mean ± SD)	117.3 ± 13.1	118.6 ± 12.7	115.2 ± 13.5	.045*
Preoperative albumin, g/L (mean ± SD)	38.5 ± 4.1	39.1 ± 3.9	37.6 ± 4.2	.013*
Surgical parameters				
Operation time, min (mean ± SD)	218.7 ± 53.6	204.5 ± 48.3	239.1 ± 55.4	<.001**
Estimated blood loss, mL (median [IQR])	300 [200–500]	250 [180–400]	400 [280–600]	<.001**
Ascites volume > 500 mL, n (%)	83 (33.9%)	37 (25.0%)	46 (47.4%)	.001**
Radical cytoreduction (R0), n (%)	162 (66.1%)	107 (72.3%)	55 (56.7%)	.015*
Intraoperative temperature management				
Operating-room temperature, °C (mean ± SD)	22.1 ± 1.3	22.5 ± 1.1	21.5 ± 1.4	<.001**
Warming method used, n (%)				<.001**
Forced-air warming only	102 (41.6%)	75 (50.7%)	27 (27.8%)	
Prewarming + forced-air warming	64 (26.1%)	49 (33.1%)	15 (15.5%)	
No warming measures	79 (32.2%)	24 (16.2%)	55 (56.7%)	

Normothermia was defined as a core body temperature ≥ 36.0°C at the end of surgery, and hypothermia was defined as <36.0°C. Preoperative hemoglobin and albumin are reported as mean ± SD; group comparisons used independent samples *t*-tests.

ASA = American Society of Anesthesiologists, BMI = body mass index; FIGO = International Federation of Gynecology and Obstetrics, IQR = interquartile range; R0 = complete macroscopic cytoreduction, SD = standard deviation.

* *P* < .05 was considered statistically significant.

** *P* < .01 highly significant.

### 3.2. Association between intraoperative thermal management and postoperative hypothermia

The incidence of postoperative hypothermia varied significantly by thermal management strategy (see Table 2). Among patients who received any form of active warming (forced-air or prewarming combined), the hypothermia rate was markedly lower compared to those without warming (25.3% vs 69.6%, *P* < .001). Stratified analysis further showed that patients who received prewarming in addition to forced-air warming had the lowest incidence of hypothermia (23.4%). Ambient operating-room temperature was also a critical factor: patients exposed to OR temperatures < 21°C had a hypothermia rate of 74.1%, whereas those in rooms ≥23°C had a significantly lower incidence (17.2%, *P* < .001). These findings suggest a strong correlation between both active warming and environmental control with postoperative thermal stability.

**Table 2:** Association between intraoperative warming strategies and postoperative hypothermia in ovarian cancer patients (n = 245).

Variable	Total (n)	Hypothermia, n (%)	Normothermia, n (%)	*P*-value
Intraoperative warming method				<.001**
Active warming (any form)	166	42 (25.3%)	124 (74.7%)	
No warming	79	55 (69.6%)	24 (30.4%)	
Warming strategy details				<.001**
Forced-air warming only	102	27 (26.5%)	75 (73.5%)	
Prewarming + forced-air warming	64	15 (23.4%)	49 (76.6%)	
Operating-room temperature (OR temp)				<.001**
<21°C	58	43 (74.1%)	15 (25.9%)	
21.0–22.9°C	129	44 (34.1%)	85 (65.9%)	
≥23°C	58	10 (17.2%)	48 (82.8%)	

Hypothermia was defined as a core body temperature < 36.0°C at the end of surgery. Active warming included any intraoperative temperature maintenance method (forced-air warming alone or in combination with prewarming). *P*-values were calculated using the chi-square test.

OR temp = operating-room temperature.

** *P* < .01 highly significant.

### 3.3. Multivariate analysis of risk factors for hypothermia

As shown in Table 3, multivariate logistic regression identified several independent predictors of postoperative hypothermia. Patients aged ≥ 60 years had higher odds of hypothermia (adjusted OR 1.89, 95% CI: 1.08–3.31, *P* = .025), as did those with BMI < 22 kg/m² (OR 1.96, *P* = .022), ASA class III–IV (OR 2.05, *P* = .020), and ascites volume > 500 mL (OR 1.97, *P* = .031). Prolonged surgery duration (≥240 minutes) and increased blood loss (≥400 mL) were also strongly associated with hypothermia, with ORs of 2.92 and 2.23, respectively (*P* < .05 for both). Importantly, intraoperative care factors emerged as the strongest predictors. Patients who did not receive active warming were nearly 4 times more likely to develop hypothermia (OR 4.12, 95% CI: 2.17–7.81, *P* < .001). Similarly, exposure to OR temperatures below 22°C significantly increased hypothermia risk (OR 2.47, *P* = .005), underscoring the impact of nursing thermal protocols and environmental control. In univariable screening, vasoactive use demonstrated a crude association with hypothermia but was not retained in the final multivariable model after stepwise selection, with attenuation after adjustment for BMI, operative time, and blood loss (see Table S1, Supplemental Digital Content, https://links.lww.com/MD/R269).

**Table 3:** Multivariate logistic regression analysis of risk factors for postoperative hypothermia in ovarian cancer patients (n = 245).

Variable	Adjusted OR (95% CI)	*P*-value
Age ≥ 60 yr (vs <60)	1.89 (1.08–3.31)	.025*
BMI < 22 kg/m² (vs ≥22)	1.96 (1.10–3.49)	.022*
ASA III–IV (vs I–II)	2.05 (1.12–3.78)	.020*
Operation time ≥ 240 min (vs <240)	2.92 (1.55–5.51)	.001**
Blood loss ≥ 400 mL (vs <400)	2.23 (1.20–4.15)	.011*
Ascites volume > 500 mL (vs ≤500)	1.97 (1.07–3.63)	.031*
No active warming (vs any warming)	4.12 (2.17–7.81)	<.001**
OR temp < 22°C (vs ≥22°C)	2.47 (1.32–4.62)	.005**

Reference groups are indicated in parentheses. Hypothermia was defined as core body temperature < 36.0°C at the end of surgery. Variables were selected for multivariate analysis based on univariate screening with *P* < .1 and clinical relevance.

ASA = American Society of Anesthesiologists, BMI = body mass index, CI = confidence interval, OR = odds ratio, OR temp = operating-room ambient temperature.

* *P* < .05 was considered statistically significant.

** *P* < .01 highly significant.

### 3.4. Postoperative outcomes associated with hypothermia

Postoperative recovery was notably impaired in patients who experienced hypothermia (see Table 4). The incidence of shivering in the PACU was more than 3 times higher in the hypothermia group (40.2% vs 12.2%, *P* < .001). These patients also had significantly higher rates of SSI (16.5% vs 6.8%, *P* = .019) and unplanned 30-day readmissions (8.2% vs 2.7%, *P* = .045). Functional recovery was delayed: the time to first flatus and ambulation was significantly longer in the hypothermia group (40.7 ± 10.1 vs 36.4 ± 9.3 hours, *P* = .002; and 28.9 ± 7.5 vs 24.6 ± 6.8 hours, *P* < .001, respectively). Furthermore, length of postoperative hospitalization was extended by more than 1 full day in hypothermic patients (9.1 ± 3.1 vs 7.9 ± 2.4 days, *P* < .001), indicating a meaningful burden on recovery time and healthcare resources.

**Table 4:** Postoperative outcomes in normothermia versus hypothermia groups following cytoreductive surgery for ovarian cancer (n = 245).

Outcome	Normothermia (n = 148)	Hypothermia (n = 97)	*P*-value
Shivering in PACU, n (%)	18 (12.2%)	39 (40.2%)	<.001**
Surgical site infection (SSI), n (%)	10 (6.8%)	16 (16.5%)	.019*
Time to first flatus, h (mean ± SD)	36.4 ± 9.3	40.7 ± 10.1	.002**
Time to first ambulation, h (mean ± SD)	24.6 ± 6.8	28.9 ± 7.5	<.001**
Length of postoperative hospital stay, d	7.9 ± 2.4	9.1 ± 3.1	<.001**
30-d unplanned readmission, n (%)	4 (2.7%)	8 (8.2%)	.045*

All outcomes were recorded prospectively in nursing charts or hospital records and verified at discharge. Hypothermia was defined as a core body temperature < 36.0°C at the end of surgery. *P*-values were calculated using chi-square test or independent samples *t*-test.

PACU = postanesthesia care unit, SD = standard deviation; SSI = surgical site infection.

* *P* < .05 was considered statistically significant.

** *P* < .01 highly significant.

## 4. Discussion

This retrospective cohort study investigated the incidence, risk factors, and clinical consequences of postoperative hypothermia in 245 ovarian cancer patients undergoing cytoreductive surgery. The overall incidence of hypothermia (core temperature < 36.0°C at the end of surgery) was 39.6%, highlighting the substantial prevalence of this preventable complication in a high-risk oncologic surgical population. Our findings identified several independent predictors of postoperative hypothermia, including advanced age (≥60 years), lower BMI (<22 kg/m²), higher ASA physical status (III–IV), ascites volume > 500 mL, prolonged operative time (≥240 minutes), increased intraoperative blood loss (≥400 mL), absence of active warming, and an operating-room temperature < 22°C.

These findings are consistent with and extend prior literature on perioperative hypothermia. Advanced age and low BMI have been previously associated with impaired thermoregulation due to diminished metabolic reserves and insulation, particularly among oncology patients undergoing extensive procedures.^[22,23]^ High ASA class has been linked to reduced physiological resilience, making patients more susceptible to hypothermic stress.^[24]^ The associations of operative time and blood loss with hypothermia are also well-documented, reflecting increased heat loss via evaporation, open exposure, and fluid shifts during prolonged and complex procedures.^[25,26]^ Our identification of ascites volume > 500 mL as a risk factor provides new insight, potentially attributable to heat loss through the evaporation of intraperitoneal fluid, which merits further investigation.

Importantly, this study confirms the protective role of active warming measures and environmental temperature control. Patients who received prewarming in conjunction with forced-air warming had the lowest incidence of hypothermia, whereas those without any warming measures were at significantly increased risk. These results support current ERAS and ASPAN recommendations advocating for proactive thermal management during surgery.^[27,28]^ Furthermore, our finding that operating-room temperatures < 22°C independently predict hypothermia aligns with prior studies and underscores the need to reevaluate institutional environmental controls.^[25]^

Hypothermia was significantly associated with worse postoperative outcomes, including a higher incidence of shivering in the PACU, increased SSI rates, delayed return of gastrointestinal function, prolonged time to ambulation, extended hospital stays, and elevated 30-day readmission rates. These findings reinforce the clinical consequences of perioperative hypothermia. Mechanistically, hypothermia induces peripheral vasoconstriction and impairs immune cell function, thereby increasing susceptibility to infection and delaying wound healing.^[4,29]^ Moreover, thermoregulatory stress may heighten postoperative discomfort and metabolic demand, contributing to delayed recovery milestones.^[30]^

This study possesses several strengths. It is among the few investigations to focus specifically on hypothermia in ovarian cancer surgery, a setting characterized by extensive surgical exposure and high fluid losses. The inclusion of environmental parameters, such as operating-room temperature and warming strategy details, adds practical value for perioperative management. Furthermore, the comprehensive analysis of both risk factors and outcomes enhances the clinical relevance of our findings.

The generalizability of our findings should also be considered. Although our cohort is limited to women undergoing ovarian cytoreductive surgery, the pathophysiology linking heat loss, anesthetic-induced thermoregulatory impairment, and hypothermia is shared across noncardiac major surgeries. Multicenter and meta-analytic data in broader surgical populations identify similar risk factors (age, low BMI, longer anesthesia/surgery time, blood loss/fluids) and report postoperative hypothermia incidences around 30% in major abdominal procedures. Therefore, the benefits of active warming and maintaining OR temperatures ≥ 22°C are likely to generalize in direction of effect, whereas the absolute incidence may be lower in surgeries with less peritoneal exposure and smaller fluid shifts than ovarian cytoreduction.

However, several limitations should be acknowledged. The retrospective, single-center design limits causal inference and generalizability. Although we adjusted for key confounders, unmeasured variables such as anesthetic depth, humidity, or fluid warming practices may have influenced outcomes. Additionally, we relied on a single time-point temperature measurement at surgical closure; continuous intraoperative temperature data could provide further insight into thermal trends. Future prospective studies, including randomized trials, are warranted to confirm these findings and assess the comparative efficacy of various warming techniques. Furthermore, by design our sample comprises female patients undergoing a single oncologic procedure, which enhances internal validity but limits direct extrapolation of incidence estimates to other surgeries; nevertheless, the modifiable thermal factors we evaluated are broadly applicable. Because no male patients were included, we cannot assess sex-related differences in thermoregulatory responses; future multicenter studies that include male cohorts are needed to evaluate external validity across sexes. Despite standardized HVAC and harmonized thermostat calibration, we cannot fully exclude unmeasured room-level micro-environmental differences (e.g., airflow patterns), which could introduce residual confounding. From a mechanistic perspective, general anesthetics (both inhalational and intravenous) are known to lower the thresholds for vasoconstriction and shivering, blunt thermoregulatory responses, and promote peripheral vasodilation, thereby facilitating core-to-peripheral heat redistribution and increasing hypothermia risk. These effects are well summarized by Rauch et al (Int J Environ Res Public Health, 2021) and are consistent with our clinical observations.^[2]^ In our cohort, anesthesia maintenance mode (volatile-based general anesthesia vs TIVA) and the use of neuraxial adjuncts were evaluated and showed no independent association with postoperative hypothermia in univariable screening (Table S1, Supplemental Digital Content, https://links.lww.com/MD/R269) and were not retained after multivariable selection. Nonetheless, because dose, timing, and depth of anesthesia (e.g., MAC or BIS targets) were not uniformly available over the study period, residual confounding by anesthetic intensity cannot be fully excluded, which we acknowledge as a limitation.^[2]^ Additionally, while vasoactive exposure was captured (any use vs none), detailed dose and timing were not uniformly available across the 9-year period, which may lead to residual confounding despite multivariable adjustment.

In conclusion, postoperative hypothermia remains a prevalent and clinically significant issue in ovarian cancer cytoreductive surgery. Risk factors span patient-related characteristics, intraoperative conditions, and environmental variables. Our findings support the implementation of comprehensive, nurse-led thermal care protocols incorporating prewarming, intraoperative forced-air warming, and ambient temperature control. Such strategies are essential for optimizing surgical outcomes and should be integrated into standardized ERAS pathways.

## Author contributions

**Conceptualization:** Lu Mo, Jing Fu, Wei Zhang, Jingyue Kuang, Tao Jiang.

**Data curation:** Lu Mo, Jing Fu, Wei Zhang, Jingyue Kuang, Tao Jiang.

**Formal analysis:** Lu Mo, Jing Fu, Jingyue Kuang, Tao Jiang.

**Investigation:** Wei Zhang.

**Methodology:** Lu Mo, Jing Fu, Wei Zhang, Jingyue Kuang, Tao Jiang.

**Resources:** Wei Zhang.

**Software:** Jing Fu, Jingyue Kuang, Tao Jiang.

**Validation:** Jing Fu, Wei Zhang.

**Writing – original draft:** Lu Mo, Jing Fu, Wei Zhang, Jingyue Kuang, Tao Jiang.

**Writing – review & editing:** Lu Mo, Jing Fu, Wei Zhang, Jingyue Kuang, Tao Jiang.

## Supplementary Material


