# Supplementary material for: Operating-room temperature and warming protocols as predictors of postoperative hypothermia in ovarian cancer surgery: A retrospective cohort study
Source: Medicine (Baltimore). 2026 Jan 30;105(5):e47452. doi: 10.1097/MD.0000000000047452 (PMC12863862; doi:10.1097/MD.0000000000047452)
Supplement: Supplementary file 1 [file medi-105-e47452-s001.docx]

**Supplementary Table S1. Univariable Logistic Regression Analysis of Risk Factors for Postoperative Hypothermia in Ovarian Cancer Cytoreductive Surgery (n = 245)**

| **Variable** | **Univariable OR (95% CI)** | **P-value** |
| --- | --- | --- |
| Age ≥ 60 years (vs < 60) | 2.01 (1.19 – 3.39) | 0.009 |
| BMI < 22 kg/m² (vs ≥ 22) | 2.08 (1.22 – 3.53) | 0.007 |
| ASA III–IV (vs I–II) | 1.86 (1.04 – 3.32) | 0.037 |
| FIGO stage III–IV (vs I–II) | 2.17 (1.19 – 3.97) | 0.011 |
| Operative time ≥ 240 min (vs < 240) | 3.25 (1.84 – 5.76) | < 0.001 |
| Blood loss ≥ 400 mL (vs < 400) | 2.58 (1.46 – 4.56) | 0.001 |
| Ascites > 500 mL (vs ≤ 500) | 2.67 (1.52 – 4.67) | 0.001 |
| Preoperative hemoglobin < 115 g/L (vs ≥ 115) | 1.42 (0.87 – 2.33) | 0.162 |
| Preoperative albumin < 38 g/L (vs ≥ 38) | 1.58 (0.94 – 2.66) | 0.083 |
| Anesthesia maintenance: volatile-based GA (vs TIVA) | 1.31 (0.76 – 2.27) | 0.327 |
| Neuraxial adjunct used (yes vs no) | 1.22 (0.71 – 2.10) | 0.473 |
| Intraoperative vasoactive use (any vs none) | 1.71 (0.96 – 3.03) | 0.069 |
| No active warming (vs any warming) | 5.49 (3.01 – 10.00) | < 0.001 |
| OR temperature < 22 °C (vs ≥ 22 °C) | 2.83 (1.63 – 4.92) | < 0.001 |

**Note:** Variables with P < 0.10 were entered into the multivariable model (see Table 3).

Preoperative hemoglobin and albumin both showed borderline associations with hypothermia in univariable testing but were not retained after multivariable stepwise selection due to attenuation when adjusted for BMI, intraoperative blood loss, and operative time (likely reflecting collinearity with nutritional and fluid-related variables).

Anesthesia-related variables (volatile-based GA vs TIVA and neuraxial adjunct use) showed no independent association with hypothermia and were not retained in the final model. Vasoactive use (phenylephrine/ephedrine; any vs none) demonstrated a crude association with hypothermia in univariable testing but was not retained after adjustment for BMI, operative time, and blood loss.
